# Preparation and Properties of Phase Change Energy Storage Composites Based on Modified Fly Ash

**DOI:** 10.3390/ma18092153

**Published:** 2025-05-07

**Authors:** Chaoheng Li, Qingchun Yu, Yong Deng, Qixiang Su, Tianlie Xiao, Yifan Sun

**Affiliations:** 1 Key Laboratory for Nonferrous Vacuum Metallurgy of Yunnan Province, Kunming University of Science and Technology, Kunming 650093, China; 18711098637@163.com (C.L.);; 2 State Key Laboratory of Complex Non-Ferrous Metal Resources Clean Utilization, Kunming University of Science and Technology, Kunming 650093, China; 3 National Engineering Research Center of Vacuum Metallurgy, Kunming University of Science and Technology, Kunming 650093, China; 4 Faculty of Metallurgical and Energy Engineering, Kunming University of Science and Technology, Kunming 650093, China

**Keywords:** fly ash, shape-stable, thermal energy storage, Al-Si alloy

## Abstract

Fly ash (FA) is a porous solid waste produced by coal-fired power plants that can be used as a carrier for solid–liquid phase change materials (PCM). Due to the disadvantages of FA, including small adsorption capacity and poor thermal performance, its application range is limited. Therefore, FA modification methods have received increasing attention. Two modification methods were used to improve the adsorption capacity of FA. After the modification experiments, the surface structure of modified fly ash (MFA) was eroded, revealing a three-dimensional porous structure. The Al/Si mass ratio of the alkali-modified sample increased from 0.67 to 1.28, and the specific surface area and pore volume increased from 3.82 m^2^/g and 0.008 cm^3^/g to 40.86 m^2^/g and 0.026 cm^3^/g, respectively. The shape-stable phase change material (SSPCM) prepared using the hybrid sintering method of Al-12Si alloy and alkali-modified fly ash (MFA-OH) exhibits excellent thermal properties and thermal cycling stability. The results showed that the heat storage density and thermal conductivity of SSPCM increased with an increase in PCM content. The thermal conductivity and latent heat of phase change in the composite with the highest latent heat of phase change in the sample were 18.24 W/(m·K) and 124.10 J/g, respectively. The optimum loading rate for the alloy is 65 wt%. After 100 thermal cycles, the latent heat and thermal conductivity of the phase change at SSPCM were 93.3% and 94.6% of the initial values, respectively. The research findings provide a feasible process for FA as a phase change carrier, and the application scope is extended.

## 1. Introduction

As a core component of modern energy systems, thermal energy storage (TES) technologies are of strategic importance for achieving carbon neutrality [1,2]. Among them, Latent Heat Storage (LHS) technology has become a key path to optimizing the efficiency of energy use due to its high energy density, reversible phase change properties, and excellent cyclic stability [3].

The efficacy of latent heat storage (LHS) systems fundamentally depends on the selection of an appropriate phase change material (PCM). Current research primarily focuses on solid–liquid PCM, which is broadly categorized into inorganic and organic materials [4]. Organic PCMs are further divided into paraffinic and non-paraffinic types (such as fatty acids and polyol) [5,6,7], while inorganic PCMs are classified into crystalline hydrated salts, molten salts, and metals or alloys [8,9,10]. Among these, aluminum and its alloys have garnered significant attention due to their high thermal conductivity, elevated heat storage density, and low subcooling—properties that render them ideal for thermal storage applications [11,12]. Nonetheless, practical applications of aluminum alloys encounter several challenges. At high temperatures, these alloys are prone to reacting with oxygen and water vapor, leading to oxide formation that compromises the performance and lifespan of the PCM. Moreover, repeated cycles of melting and solidification at elevated temperatures can alter the microstructural integrity of the material, thereby affecting the reliability of its thermal properties. Prolonged exposure to high temperatures may also degrade the mechanical properties of the alloys. These issues collectively diminish the service life of aluminum alloys and limit their broader application in various fields [13,14,15].

Encapsulation of phase change materials is an effective solution to the above problem. Currently, the encapsulation of Al and its alloy PCMs is categorized into micro and macro by size [13]. The available literature shows that the research on microencapsulation of phase change materials has made significant progress. For example, Wang et al. [16] prepared SnBi58 alloy microencapsulated phase change materials (MEPCMs) with high thermal reliability by using a “two-layer coating at the expense of the inner layer” method, which constructed thermal expansion voids to accommodate the volume expansion of the PCM. The latent heat of the MEPCM/ceramic composites was increased by 30.24 J/g, and thermal conductivity reached 2.853 W/(m·K). Han [17] proposed a method for the preparation of Al-12Si@Al_2_O_3_ phase change alloy particles by pressurized steam etching and high-temperature heat treatment using Al-12Si as a phase change material. The Al-12Si@Al_2_O_3_ capsule retained a high latent heat of 261.8 J g^−1^ after 300 thermal cycles. T. Nomura et al. [18] conducted an extensive study on the preparation of Al-Si alloy microcapsules by the hydrothermal method combined with thermal oxidation. The durability of Al-12Si@Al_2_O_3_ microcapsules exceeded 3000 thermal cycles with latent heat ranging from 186 J/g to 347 J/g, which showed excellent durability and high heat storage capacity. Although microcapsules have superior thermal properties and application potential, there are constraints such as complex preparation technology and high cost. Compared to microencapsulation, shape-stabilized microencapsulation is simpler, while maintaining stable performance in extreme operating environments.

In response to the need for corrosion protection of aluminum-based phase change materials at high operating temperatures, Jacob et al. [19] systematically reviewed the metal-encapsulated housing materials and pointed out that ceramic materials (especially Al_2_O_3_) can significantly enhance the reliability of PCM encapsulation systems due to their excellent resistance to liquid metal corrosion and high thermal conductivity. Fukahori’s team [20] innovatively designed a macro-encapsulation system with a stress-buffering structure of Al_2_O_3_ ceramics: sintered Al_2_O_3_ cylinders were used to encapsulate Al-25Si alloys, and through the reserving of the volume-expansion void, the structural integrity of the composite is maintained after 100 thermal cycles. In terms of structural optimization, Lao et al. [21] developed SiCw/Al_2_O_3_ honeycomb ceramics with hexagonal holes as the shell material, and utilized the multistage pores of the honeycomb structure to achieve efficient loading of PCM. Experiments show that the composite material has an enhanced thermal storage capacity of 114% and no interfacial peeling after 100 thermal shocks. The current research trend indicates that porous carrier technology can reduce the risk of PCM leakage through the capillary force confinement effect, but traditional carbon-based materials (e.g., expanded graphite, carbon nanotubes [22,23]) are difficult to apply on a large scale due to the high cost of raw materials. Fly ash (FA), as an industrial solid waste with an annual production of more than 10^9^ tons globally, opens up a new path for low-cost encapsulation material development by virtue of its multistage pore structure and high temperature resistance [24,25]. Our previous study showed that alkali-treated FA (MFA) could enhance the pore volume from 0.008 cm^3^/g to 0.085 cm^3^/g, which lays the structural foundation for the subsequent efficient loading of PCM.

In this paper, an untreated power plant FA was modified to improve the adsorption rate of fly ash. Comparative experiments were carried out to demonstrate the effectiveness of fly ash modification. The results showed that the pore structure of the modified treated MFA was richer, especially the pore volume of the alkali-treated MFA was larger. The Al-12Si/MFA composite phase change thermal storage material was prepared by the high-temperature sintering method using MFA as the support material and Al-12Si as the phase change material. The thermal storage performance and thermal stabilization capability of the composite materials were evaluated. Finally, the reliability of the developed composites was demonstrated by 100 heating and cooling cycles. This work provides ideas on how to effectively deal with power plant solid waste and its application in energy storage.

## 2. Materials and Methods

The Al-12Si alloy was provided by AVIC Mighty Powder Metallurgy Technology Co., Ltd. (Beijing, China) Sodium hydroxide (NaOH, with 99% purity) and polyvinyl alcohol were obtained from Sinopharm Chemical Reagent Co., Ltd. (Shanghai, China) Hydrochloric acid (HCl, Concentration 37%) was provided by the Beijing Reagent Company (Beijing, China). Fly ash (FA) samples collected from Henan Gongyi Cogeneration Power Plant were classified as Class F [26].

**Figure 1:**
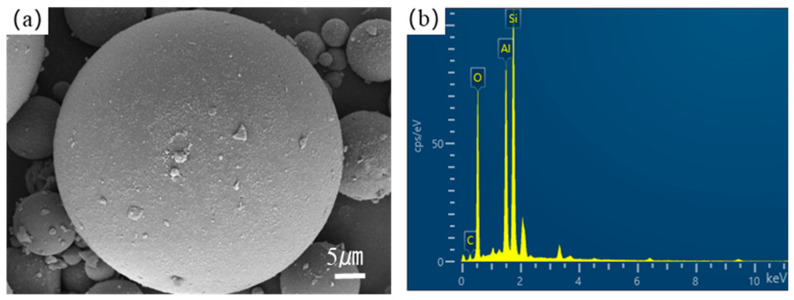
(**a**) SEM image and (**b**) EDS spectrum of fly ash.

Phase analysis via XRD showed that the main crystalline phases were mullite and quartz, and trace amounts of hematite (Fe_2_O_3_) and amorphous carbon were detected. Scanning electron microscopy showed, as seen in Figure 1, that the untreated FA particles exhibited a monodisperse spherical morphology with a smooth surface and limited porosity [27].

### 2.1. Fly Ash Pretreatment 

The raw FA was subjected to decarbonization pretreatment in a muffle furnace. A precisely weighed 50 g FA sample was transferred into an alumina crucible (99.6% purity, 100 mL capacity) and calcined at 600 °C (heating rate 5 °C/min) for 2 h under ambient atmosphere. Subsequent natural cooling to room temperature facilitated the complete oxidation of residual carbon and sulfur compounds. A certain amount of FA was weighted into the crucible, then the dry pot was put into the resistance furnace, roasted at a temperature of 600 °C for 2 h, and then cooled to room temperature, so that the unburned carbon and sulfur in the fly ash was fully oxidized, and the gas was fully discharged.

### 2.2. Acid-Alkali Modification Protocols

**Figure 2:**
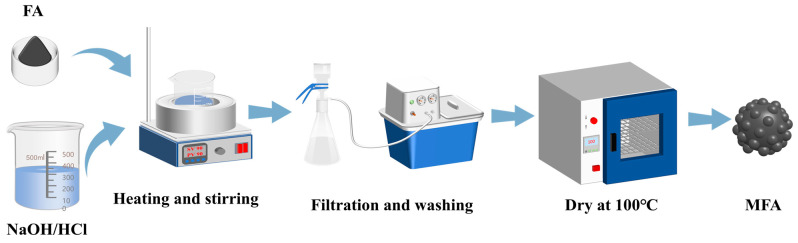
Schematic flowchart of modification.

Three distinct modification strategies were systematically investigated, as illustrated in Figure 2:

(1) Thermal Activation (TA-FA):
  The pretreated FA was directly calcined under identical conditions (600 °C, 2 h) for comparative analysis.
(2) Acid Leaching Modification (AL-FA):
  1. Prepared hydrochloric acid solution with a certain concentration gradient (0.5–2 mol/L, 0.5 mol/L increments);
  2. Mixed the two in a beaker at a liquid/solid ratio (10:1);
  3. Conducted continuous magnetic stirring at 90 °C for 2 h;
  4. Performed vacuum filtration followed by neutralization washing;
  5. Dried obtained FA at 70 °C for 24 h.
(3) Alkaline Modification (AM-FA):
  1. Prepared sodium hydroxide solution with a certain concentration gradient (0.5–2 mol/L, 0.5 mol/L increments);
  2. Established liquid/solid system (10:1 *v*/*w* ratio) in a PTFE reaction vessel;
  3. Conducted continuous magnetic stirring at 90 °C for 2 h;
  4. Performed vacuum filtration followed by neutralization washing;
  5. Post-dried at 70 °C until constant mass (Δm < 0.1% over 2 h).

### 2.3. Preparation of Al-12Si/FA SSPCM

Al-12Si/MFA phase change heat storage materials were prepared by using a high-temperature sintering method. As shown in Figure 2, Al-12Si and MFA were mixed in a certain proportion and ground thoroughly, and PVA solution with a mass fraction of 5% was used as a binder to press the mixture into a circular sheet with a diameter of 20 mm and a height of 5 mm. The circular sheet was then dried thoroughly and placed in a resistance furnace for high-temperature sintering at 950 °C with a holding time of 2 h to prepare the SSPCM.

In order to assess the effectiveness of the alloy in the MFA, an effective alloying rate *R* was introduced and calculated as shown in Equation (1):

(1) $$R=\frac{{∆H}_{real}}{{∆H}_{0}\times w}\times 100\%$$

- *R*: the efficiency of A in Al-12Si/MFA (%);
- ${\Delta H}_{real}$: composite PCM latent heat (DSC measurement);
- Δ*H*_0_: latent heat of pure Al-12Si (DSC measurements);
- *w*: mass fraction of initial alloy in SSPCM.

Al-12Si alloy powder is a phase change medium, with composite embedded in the FA-fired ceramic matrix skeleton; the pore structure in the matrix provides space for accommodating the phase change medium, when the phase change medium reaches the phase change temperature in the liquid state, due to the role of surface tension and capillary force, it can still be stabilized in the ceramic matrix. Due to the content of the phase change medium and sintering conditions, when the capillary force and surface tension are not enough to adsorb the phase change medium, it will lead to liquid phase change medium exudation and deformation of the matrix. Therefore, a reasonable proportion of phase change medium can maximize the latent heat of phase change and, at the same time, ensure that SSPCM has a certain mechanical strength and does not leak during operation.

### 2.4. Characterization

The content of inorganic oxides in FA was determined via X-ray fluorescence spectrometry (XRF, RIGAKU ZSX Primus, Rigaku, Tokyo, Japan) before and after the modification, and the morphology and microstructure of FA were observed by scanning electron microscopy (SEM) and X-ray energy spectrometry (SEM-EDS, TESCAN MIRA, Tescan, Brno, Czech Republic) with an accelerating voltage of 5 KV before and after modification. X-ray diffraction (XRD, Rigaku SmartLab SE, Rigaku, Tokyo, Japan) was used to study the physical phase composition of FA, MFA, and HTSSPCM with scanning angles of 10°~90° and a scanning speed of 2°/min. The N_2_ adsorption–desorption isotherms of FA and MFA were determined by a fully automated specific surface and porosity analyzer at 77 K. The materials needed to be dehydrated at 200 °C for 10 h. Specific surface area was calculated using the Brunauer–Emmet–Teller (BET) [28] method using adsorption and desorption isotherms. The total pore volume was obtained from the maximum amount of N_2_ adsorbed at a partial pressure. (BET, Micromeritics ASAP 2460, Micromeritics, Norcross, GA, USA). The thermal properties of the materials were measured by Differential Scanning Calorimetry (DSC, TA, New Castle, DE, USA), with the temperature being increased at a rate of 10 °C/min under a stream of nitrogen. Thermal conductivity was determined by a thermal conductivity meter (Hot Disk TPS2500S, Hot Disk, Gothenburg, Sweden) [29]. An electronic universal testing machine (5969, INSTRON, Norwood, MA, USA) was used to test the compressive strength of the samples at room temperature with a crosshead speed of 0.5 mm∙min^−1^.

## 3. Results and Discussion

### 3.1. Chemical Composition and Characterization of FA/MFA

**Figure 3:**
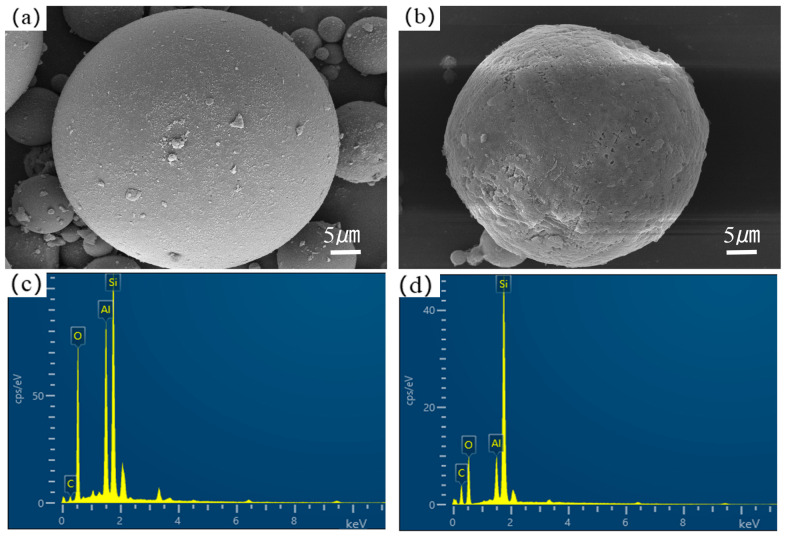
SEM images of (**a**) FA and (**b**) FA-H; (**c**,**d**) corresponding EDS patterns.

**Figure 4:**
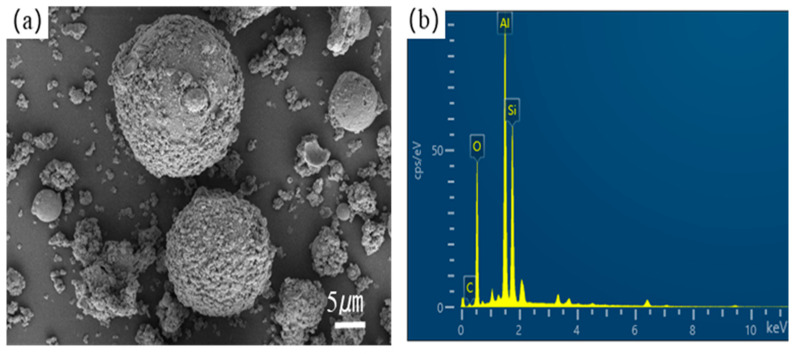
SEM images of (**a**) FA-OH and (**b**) corresponding EDS pattern.

The chemical composition and morphological evolution of fly ash (FA) carriers fundamentally govern the performance of shape-stabilized phase change materials (SSPCM). Scanning electron microscopy (SEM) analyses (Figure 3 and Figure 4) revealed distinct surface restructuring during modification processes. Complementary X-ray fluorescence (XRF) spectroscopy (Table 1) quantified compositional variations, confirming aluminosilicate dominance (SiO_2_: 51.10 wt%, Al_2_O_3_: 26.43 wt%) in both pristine FA and modified FA (MFA), consistent with Class F fly ash characteristics.

### 3.2. Structural and Physical Analysis of FA, MFA

The specific surface area and total pore volume of the modified fly ash increased with the concentration of the modified solution. When the solution concentration exceeded 2 mol/L, the specific surface area of the two modified FAs increased slowly, while the total porosity decreased significantly.

As shown in Table 2, the specific surface area and total porosity of the modified fly ash increased significantly with the increase in the modified solution concentration, and the specific surface area of the modified fly ash increased slowly by continuing to increase the modified solution concentration, but the total porosity of both decreased.

This was due to the corrosion of the modified fly ash surface. As a result, more closed pores were opened, and the surface of fly ash particles showed a rough and porous morphology, which greatly increased the specific surface area of fly ash. The pore volume appeared as an inflection point with an increase in the concentration of the modified solution. This is due to the strong acid modification process; the high concentration of hydrochloric acid will cause the fly ash to be excessively corroded, so that the skeleton structure of fly ash is deformed or even collapsed, leading to the destruction of some pores and the reduction in the total pore volume [30]. Similarly high concentration of sodium hydroxide solution will seriously damage the outer shell of fly ash particles, causing the internal amorphous vitreous to be dissolved, which in turn destroys the integrity of the skeleton of fly ash particles [31].

**Figure 5:**
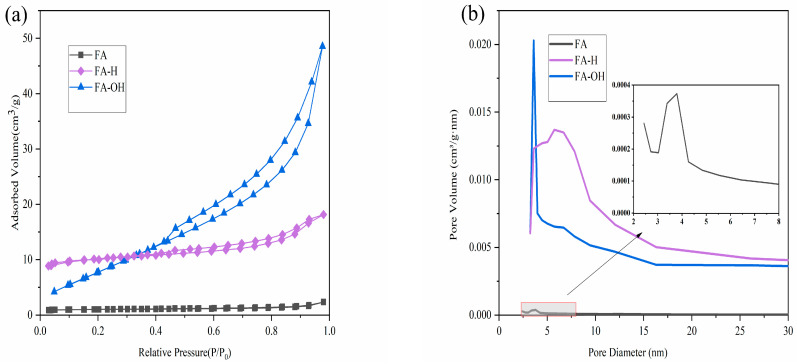
(**a**) Nitrogen adsorption–desorption isotherms and (**b**) pore distribution of FA, FA-H, and FA-OH.

As shown in Figure 5, the pore size of hydrochloric acid-modified fly ash was mainly distributed in 4–15 nm, and the pore volume increased. Compared with the pre-modification, the pore size increased significantly, and larger pores appeared. However, as a matrix material, when the pore volume is certain, the pore size is too large, leading to a decrease in the capillary force, and when the phase change material enters it, it undergoes a phase change, the capillary tension. Insufficient pressure will make the phase change material in a molten state flow out of the pore, and leakage occurs. At the same time. The aluminum–silicon ratio of fly ash increased after modification with sodium hydroxide, and the metal oxides (Na_2_O, MgO) in the composition were retained, which can effectively improve the thermal conductivity of the composite phase change material. Therefore, the modified fly ash used in the subsequent experiments was produced by using a 2 mol/L sodium hydroxide solution as the modification solution.

**Figure 6:**
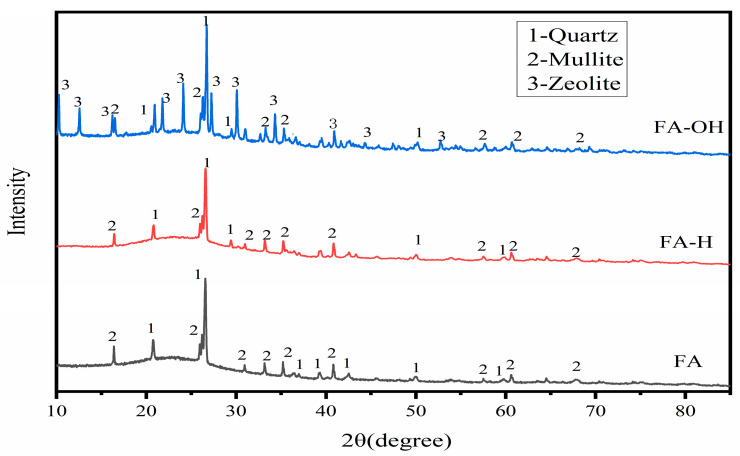
XRD patterns of FA, FA-H, and FA-OH.

The structural changes in FA before and after modification were detected and analyzed by XRD, and the XRD spectra of FA and MFA are shown in Figure 6. For FA, the 2θ diffraction peaks located at 16.4°, 26°, 33.2°, 35.2°, and 40.8° are characteristic diffraction peaks of mullite, while the 2θ diffraction peaks located at 20.8°, 26.6°, and 50.0° are absorption peaks of quartz. The relatively broad diffraction peaks between 15° and 30° indicate the presence of a large number of other amorphous silica specular bodies in the FA. This suggests that the fly ash consists mainly of mullite, quartz, and a large amount of amorphous siliceous vitrinite. Compared with the diffractograms of FA, the above characteristic peaks also appear in the diffractograms of FA-H. No new phases are produced, and only the intensities of the diffraction peaks are slightly changed. This indicates that quartz and mullite are basically insoluble in hydrochloric acid solution, but there is no obvious change in the diffraction peaks between 15° and 30° in FA-H, and a large amount of amorphous material still exists.

In addition to the presence of quartz and mullite phases in the diffraction pattern of alkali-modified fly ash, the diffraction peaks at 2θ 10.4°, 12.6°, 21.8°, 24.1°, and 27.2° belong to zeolite [32], which suggests that zeolite phases are incorporated in the chemical composition of modified fly ash, which can be used as a good sintering aid. The broad diffraction peaks appearing between 15° and 30° were significantly reduced after modification, indicating that the main effect of sodium hydroxide-modified fly ash is to chemically dissociate the SiO_2_ on the surface of fly ash with alkali, and this reaction destroys the surface structure of fly ash, which can significantly increase its specific surface area. The results obtained are consistent with the EDS spectra and XRF analyses.

### 3.3. Maximum Loading Ratio of FA, MFA on Al-12Si Alloy

**Figure 7:**
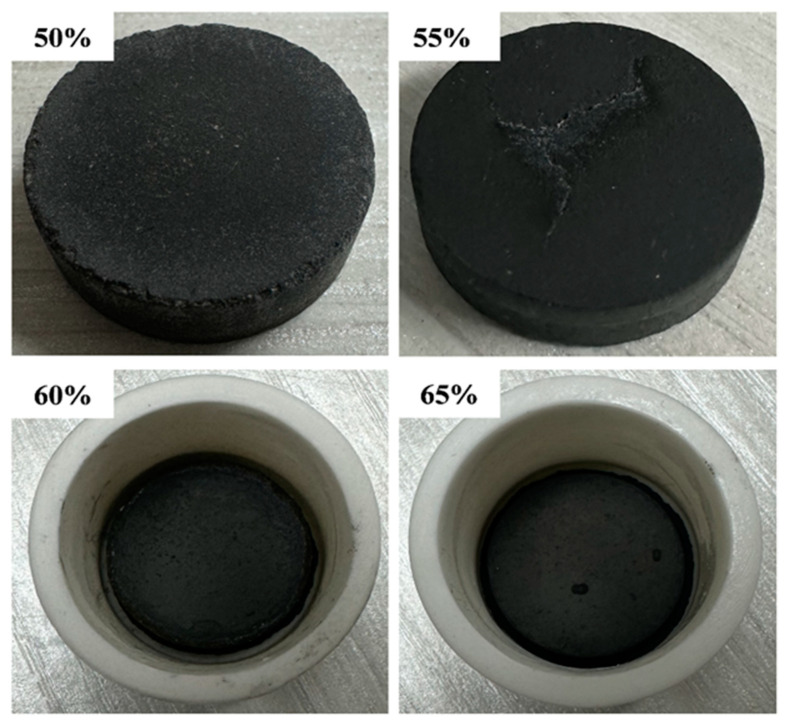
Photographs of Al-12Si/FA SSCPCM with different ratios of Al-12Si alloy.

The higher the mass ratio of Al-12Si alloy to carrier material, the higher the latent heat of phase transition of SSPCM, but there should be no leakage and continuous heat absorption when the phase transition temperature is reached during the change in Al-12Si alloy from solid to molten state. Figure 7 shows the SSPCM prepared with different percentages (Al-12Si alloy: 50 wt%, 55 wt%, 60 wt%, 65 wt%) of unmodified FA Al-12Si alloy.

**Figure 8:**
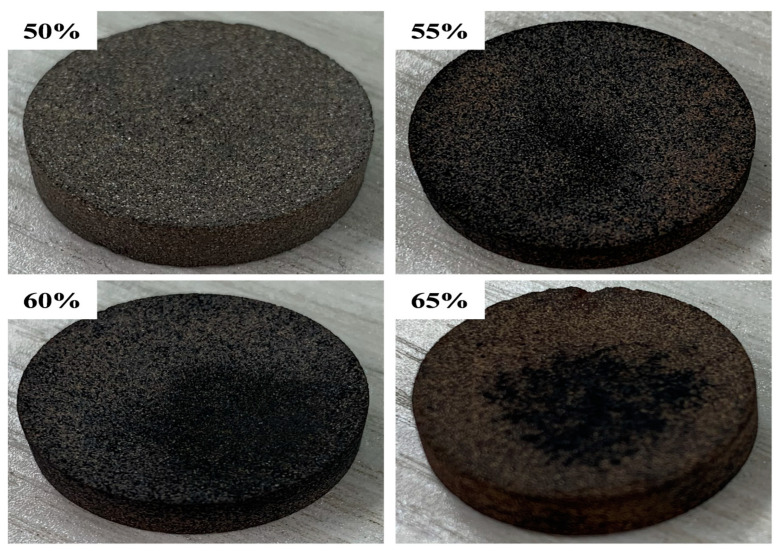
Photographs of Al-12Si/FA-H HTSCPCM with different ratios of Al-12Si alloy.

**Figure 9:**
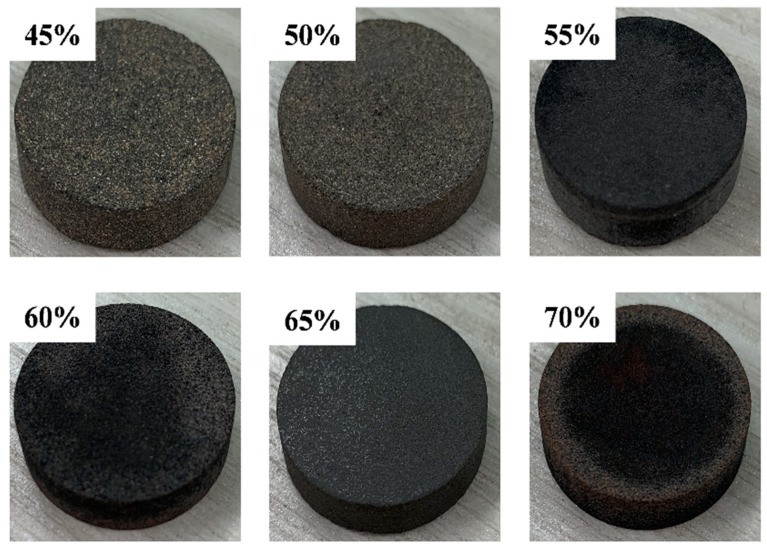
Photographs of Al-12Si/FA-OH HTSCPCM with different ratios of Al-12Si alloy.

Figure 8 and Figure 9 show the SSPCM prepared with different proportions of Al-12 Si alloy (Al-12 Si alloy: 50 wt%, 55 wt%, 60 wt%, and 65 wt%) and MFA, respectively. Leakage from the bottom of the alkali-modified composites occurs only when the proportion of Al-12 Si alloy reaches 70%. This is due to the higher content of Al-12 Si alloy. During the solid–liquid phase change process, the gravity and surface tension of the phase change medium are greater than the capillary force and surface tension provided by the ceramic matrix skeleton, and the phase change medium in the molten state aggregates downwards. The use of FA-H as the ceramic matrix carrier can effectively improve the loading capacity, but due to its large pore size, it will lead to a decrease in tension and cannot carry PCM, whereas the zeolite phase FA-OH generated in the modification process has a smaller pore size and a larger pore volume, which can further improve the adsorption capacity of the matrix and ensure that the matrix has a better loading capacity. Therefore, high-temperature sintering experiments show that the maximum loading capacity of FA-OH can reach 65 wt%. It is a more ideal matrix material in ceramic matrix.

### 3.4. Mechanical Performance Analysis

In vertically oriented packed-bed thermal energy storage systems, the structural integrity of the shape-stabilized composite phase change material (SSPCM) is critical to withstand operational compressive stresses and ensure long-term durability. As shown in Table 3, the compressive strength of Al-12Si alloy/ceramic composites is positively correlated with the alloy content, which decreases above a critical point. This increase in mechanical properties results from two synergistic mechanisms:

1. Microstructural densification: The superior fluidity of molten Al-12Si allows it to efficiently penetrate into the fly ash matrix through capillaries. Compared with the low-alloy composites, the alloy content increases from 50 wt% to 65 wt%, the porosity decreases from 17.8% to 11.5%, and the complex and irregular pores gradually disappear. Stress transfer is more uniform, and the risk of a localized rupture is reduced [33,34]. However, the main reason for the decrease in strength of the composites as the alloy content increases is the large difference in the coefficient of thermal expansion between the ceramic matrix and the alloy reinforcement [35]. As the composites are working, the stresses generated by the thermal expansion of the two tend to crack the material, shortening the service life of the phase change material.
2. Interfacial strengthening [35]: The increase in working temperature enhances the fluidity of the alloy, generating internal vapor pressure, which drives the liquid phase flow. The penetration of the alloy melt between pores during the sintering process forms mechanical interlocking [36] (mechanical interlocking). To achieve the purpose of interfacial enhancement. Meanwhile, the composite of porous ceramics and Al-Si alloy through high-temperature sintering significantly improves the flexural strength through the generation of the Al-Si-O glass phase [37] by the Si element at the interface. This process promotes the interfacial bonding between the metal phase and the ceramic matrix, resulting in a 4.5% increase in the density of the composite compared to the ceramic matrix (ρ = 1.74→1.82 g/cm^3^).

The resulting composite structure exhibits an optimized stress distribution, with the continuous alloy network carrying axial loads and the ceramic matrix resisting radial deformations. This dual-phase reinforcement mechanism operates reliably under cyclic compressive stresses of up to 50 MPa, meeting the design requirements for industrial pilot-scale thermal storage applications.

**Figure 10:**
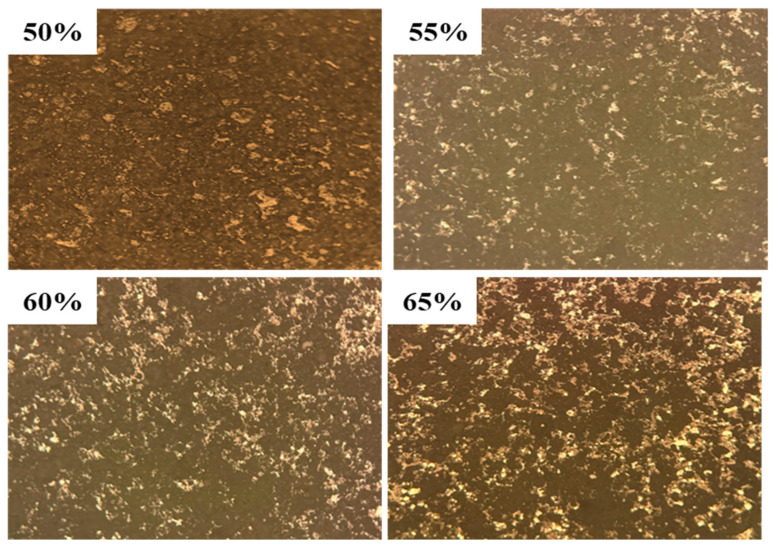
Metallographic micrographs of composite phase change materials with modified fly ash and different Al-12Si alloy ratios (100×).

Figure 10 shows a metallurgical photomicrograph (100× magnification) of a modified fly ash-based composite phase change material (SSPCM) containing different Al-12Si alloy ratios (50–65 wt%). Microstructural analysis revealed two distinct phases: a metallic luster region corresponding to the Al-12Si alloy and a continuous gray-black ceramic matrix formed using sintered modified fly ash. The alloy content directly affects the microstructure evolution. At 50 wt% alloy loading, sparse metal areas coexist with a wide porosity (17.8%). Alloy additions of up to 65 wt% allow for uniform metallic phase distribution and a reduced porosity of 11.5%. This improvement in microstructure enhances the interfacial bonding, which increases the compressive strength of the material.

### 3.5. Thermal Performance Analysis

Thermophysical properties, especially the latent heat and temperature of phase transition, are key evaluation metrics for high-temperature shape-stabilized composite phase change materials (SSPCMs). Differential scanning calorimetry (DSC) analysis reveals the thermal properties of Al-12Si alloy powder. The phase transition temperature of the alloy is 580 °C, and the latent heat of phase transition is 391.7 J/g.

**Figure 11:**
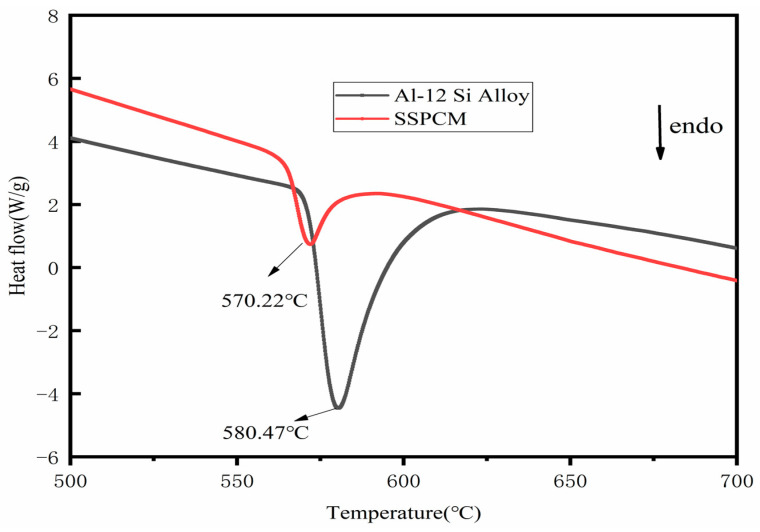
DSC curves of Al-12Si/FA-OH SSPCM (Al-12Si alloy: 55 wt%), Al-12Si alloy powder.

DSC evaluation of the composites (shown in Figure 11) highlights the performance of the materials. The Al-12Si/FA-OH composite (55 wt% alloy) shows improved performance, with a latent heat of 124.10 J/g at 581.59 °C, reflecting a 57.6% retention efficiency. The reduced latent heat of the composites is mainly attributed to the pore structure of the modified FA-OH matrix, where Al in the Al-12Si alloy oxidizes to form Al_2_O_3_, which reduces the effective phase change material (PCM) fraction by 12–18%, depending on the sintering conditions. The formation of Al_2_O_3_/SiO_2_ oxide film on the surface of the Al-12Si alloy inhibits further reactions, but the volume expansion leads to stress generation. As shown in Table 4, the mass of the composite talent material increases slowly with the number of cycles.

As shown in Table 4, pure Al-12Si has high conductivity (101.41 W/(m·K)), while the composite material reaches 18.24 W/(m·K), which is a 5.6-fold improvement over the fly ash matrix (0.18 W/(m·K)). Micropores provide capillary confinement for the molten alloy, while mesopores (5–50 nm) facilitate alloy–ceramic heat transfer [38]. Despite incomplete pore filling, the thermal storage efficiency of this composite is higher than that of conventional inorganic salt PCM, demonstrating the feasibility of metal–ceramic composites for high-temperature (500–800 °C) energy storage applications.

**Figure 12:**
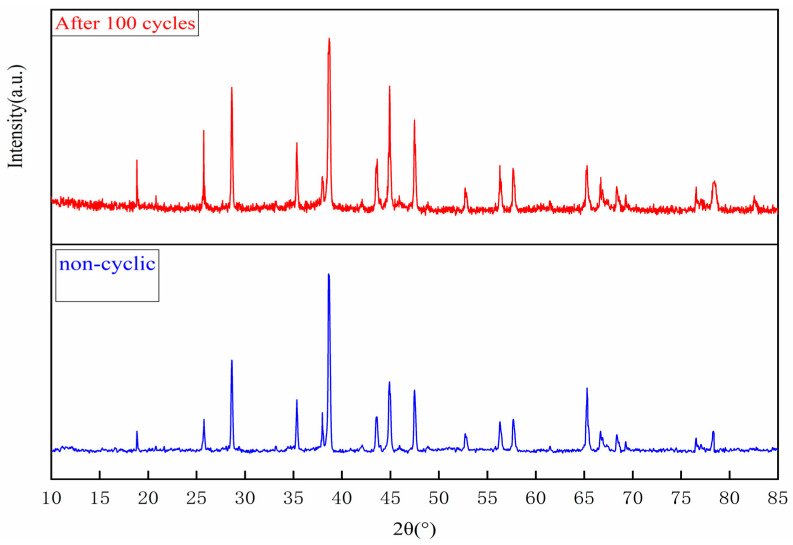
XRD patterns of composite phase change materials before and after hot and cold cycling.

Cyclic thermal stability tests revealed key performance characteristics of the composite phase change material. XRD analysis (Figure 12) showed that the phase composition remained stable after 100 thermal cycles, with no crystalline phases. However, due to partial oxidation of the exposed alloy region on the surface, the peak metallic aluminum strength decreased by 12% (2θ = 38.5°), with a corresponding 8% increase in the Al_2_O_3_ feature (2θ = 35.1°), which demonstrates the gradual oxidation of the surface.

As shown in Table 5, the analysis of the mass evolution reveals a nonlinear weight gain curve: a 1.9% increase in mass during the first 20 cycles (3.71→3.78 g), followed by a stabilization (3.82 g after 100 cycles, for a total weight gain of 2.96%). This behavior reflects two-stage oxidation kinetics: a rapid transformation of the surface alloy into a protective Al_2_O_3_ shell, followed by diffusion-limited oxidation kinetics as the ceramic matrix restricts oxygen penetration into the encapsulated alloy.

The SSPCM remained dimensionally stable throughout the testing process, thus confirming the effectiveness of the matrix encapsulation. After 100 cycles, the composite retained 93% of its latent heat of phase change, demonstrating superior cycling durability.

## 4. Conclusions

This work developed a novel ceramic-based high-temperature shape-stabilized composite phase change material (SSPCM) by compositing modified fly ash (MFA) with Al-12Si alloy. The high-temperature shape-stable phase change composites were prepared by systematically investigating the fly ash (FA) modification process and optimizing the alloy ratio. The main conclusions are as follows:

### 4.1. FA Modification Mechanisms

Alkali modification disrupted Si-O-Si/Al networks, enhancing surface reactivity and porosity (specific surface area increased by 68% to 40.86 m^2^/g).

Acid modification removed amorphous impurities while preserving the aluminosilicate framework, improving adsorption capacity by 42%.

### 4.2. Superior Thermal Performance

The optimized SSCPCM (55 wt.% Al-12Si/MFA) has a latent heat of 124.10 J/g and a thermal conductivity of 15.10 W/(m·K). The cyclic stability test showed that after 100 thermal cycles, the latent heat retention was 93.3%, the thermal conductivity retention was 93.3%, and the mass increased only 2.96%.

### 4.3. Microstructural and Chemical Stability

Metallographic analysis confirmed that Al-12Si was uniformly distributed in the hierarchical pore structure (1–5 nm micropores, 5–20 nm mesopores) of the MFA, with an alloy content of 55 wt% and no leakage. XRD confirmed the physical interactions between phases, and no deleterious chemical reactions occurred during sintering or cycling.

## 5. Further Research

Although alloy/ceramic composite phase change materials have potential in terms of heat storage density and cost, breakthroughs in interfacial compatibility, stress management, and preparation losses are still needed. In the future, it is necessary to optimize the preparation process by combining multiscale simulation (e.g., molecular dynamics to optimize the interface) and pretreatment or replacement of phase change materials to promote their practical applications.

## Figures and Tables

**Table 1:** The content of oxides in fly ash obtained via different treatment methods (%).

Sample	SiO_2_	Al_2_O_3_	CaO	Fe_2_O_3_	MgO	Na_2_O	Loss on Ignition
FA	51.10	26.43	3.96	4.65	0.79	0.72	7.74
FA-H	59.97	23.42	1.60	1.26	0.51	0.55	6.86
FA-OH	39.30	31.65	4.18	4.60	0.87	8.69	7.35

**Table 2:** Specific surface area and total pore volume of fly ash after different concentrations of acid and alkali modified solutions.

Concentration (mol/L)	HCl/Specific Surface Area (m^2^/g)	HCl/Total Pore Volume (cm^3^/g)	NaOH/Specific Surface Area (m^2^/g)	NaOH/Total Pore Volume (cm^3^/g)
0.5	7.35	0.012	9.31	0.023
1	18.26	0.017	19.23	0.046
1.5	25.34	0.023	31.24	0.069
2.0	34.06	0.026	40.86	0.085
2.5	36.47	0.027	41.86	0.073
3.0	37.51	0.026	42.75	0.064

**Table 3:** Porosity and compressive strength of composite phase change materials with modified fly ash and different Al-12Si alloy ratios.

Al-12Si Alloy Content (wt%)	Compressive Strength (MPa)	Porosity of Composites (%)
50	46.4	17.8
55	51.5	15.4
60	47.1	13.1
65	44.3	11.5

**Table 4:** Thermal conductivity of MFA, Al-12Si, and Al-12Si/FA-OH SSPCM.

Item	FA	Al-12Si	Al-12Si/FA-OH SSPCM
thermal conductivity (W/(m·K))	0.18	101.45	18.24

**Table 5:** Variation in the mass of a composite phase change material with the number of cycles.

Number of Cycles	0	20	40	60	80	100
Mass (g)	3.71	3.78	3.79	3.81	3.82	3.82

## Data Availability

The raw data supporting the conclusions of this article will be made available by the authors on request.
